# Oristry: Another Name

**Published:** 1894-09

**Authors:** Jacob L. Williams

**Affiliations:** Boston


### Domestic Correspondence.

### ORISTRY: ANOTHER NAME.

To the Editor:

Sir,—I think the International Dental Journal is the first journal to recognize the fact, as stated in its editorial on page 476 of the July number, that "the term dentistry has ceased to represent modern thought and practice, and must therefore give way to a newer word."

This matter of nomenclature for the advanced practice was discussed in 1880, by a small professional club, and both the Greek word, stomatology, and the Latin word, oristry, were suggested.

The word oristry is formed from the Latin os, oris, in the same way that the word dentistry is formed from dens, dentis; but the latter means only the teeth, while oris comprehends the mouth, including the teeth, of course. And it is the care of the health of the mouth that now constitutes the best practice with the large body of educated men in the profession.

Preferring the word oristry as being the more pleasant in sound, and easier for the laity to understand, I have adopted it since 1880.

I will add an extract from my address to our section in the American Medical Association, at Nashville, Tenn., in May, 1890. I said, "In earlier years there were only a few qualified practitioners who devoted their knowledge and skill to the treatment of the whole oral cavity, while the larger number gave their attention simply to the teeth, and so the specialty was called dentistry. But at this day, when knowledge of the principles of medicine and surgery is more general, and more commonly made available in the treatment of the whole oral cavity and with reference to its influence on the health of the system, the old term seems too limited. And what now would be a more proper and comprehensive name for the specialty, so practised, than the word oristry?

Jacob L. Williams.

Boston, July 10, 1894.

